# A case of helix-fixation leadless pacemaker dislodgment and retrieval: The importance of achieving appropriate postimplant impedance

**DOI:** 10.1016/j.hrcr.2024.02.019

**Published:** 2024-03-07

**Authors:** Faris Haddadin, Gilad Birnbaum, Laith Alhuneafat, Ahmad Jabri, Owais Ulhaq, Irakli Giorgberidze, Hamid Afshar

**Affiliations:** ∗ Department of Cardiovascular Medicine, University of Minnesota, Minneapolis, Minnesota; † Section of Cardiology, Baylor College of Medicine, Houston, Texas; ‡ Department of Cardiology, Henry Ford Hospital, Detroit, Michigan; § Michael E. DeBakey Veterans Affairs Medical Center, Section of Cardiology, Baylor College of Medicine, Houston, Texas

**Keywords:** Leadless pacemaker, Dislodgment, Retrieval, Pacemaker, Snaring, Helix-fixation

**Key Teaching Points**

• AVEIR leadless pacemaker (Abbott, Chicago, IL) is a relatively new device to the market that has proven safety and efficacy in published literature.
• Device dislodgment is considered rare, but with growing experience in real-life implants, approaches and safety checkpoints for stable implantation are being further discovered and reported.
• In our reported case, the risk of dislodgment potentially increased owing to suboptimal intraprocedural postimplantation impedance.
• A simple goose-neck snare can be used to safely capture and retrieve the AVEIR^TM^ leadless pacemaker over its docking button.

## Introduction

The AVEIR^TM^ (Abbott, Chicago, IL) helix-fixation leadless pacemaker (LP) system received Food and Drug Administration approval in April 2022. The device has proven safety and efficacy in the LEADLESS II clinical trial.^1^ We present a 93-year-old man who was admitted to the hospital with symptomatic sinus bradycardia. After shared decision-making, the patient underwent AVEIR VR leadless pacemaker placement. Immediately after implantation, the device had dislodged. We detail a stepwise approach for retrieval and reimplantation, highlighting potential reasons for premature device migration and key points for successful implantation.

## Case report

A 93-year-old man, with a history of hypertension and mild dementia, presented with recurrent syncope. Vital signs were normal, except for a heart rate of 54 beats per minute. An electrocardiogram showed sinus bradycardia (43 beats per minute) with nonspecific ST-T wave changes. Transthoracic echocardiogram revealed a preserved ejection fraction with no significant abnormalities. After ruling out reversible causes, shared decision-making with the family and patient led to the choice of LP implantation over a transvenous system, considering the patient’s advanced age, cognitive impairment, symptomatic bradycardia, history of falls, and recurrent syncope. The patient was taken for AVEIR VR LP right ventricular endocardial pacemaker implantation in the electrophysiology lab.

**Figure 1:**
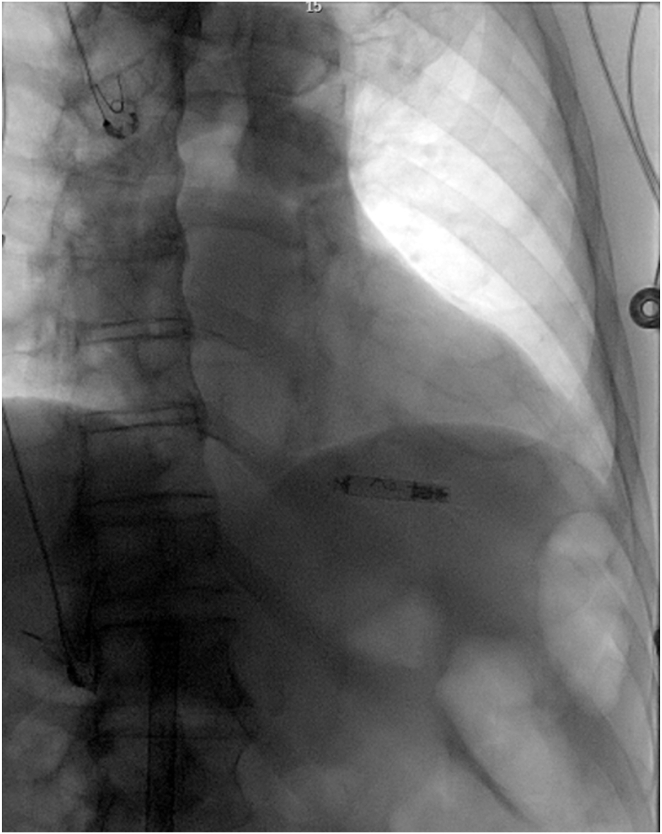
Initial leadless pacemaker device deployment.

The device was meticulously advanced into the right ventricle using standard techniques, employing a stiff wire, sequential groin dilatation, a 27F sheath, and the delivery system. Iodine contrast dye facilitated the identification of the interventricular septum. Predeployment, R-wave sensing measured 6 mV with evidence of injury. Deployment followed the standard helix fixation technique, placing the device in a mid-interventricular septal position under fluoroscopy guidance. The device was securely fixed with 1 ½ turns, achieving appropriate injury current, acceptable sensing, and a pacing threshold of 2.0 V at 0.5 ms. Fixation validation involved deflecting the delivery catheter 30–45 degrees while observing device movement, resulting in an impedance increase from 300 Ω to 320 Ω after a suitable waiting period. The device was released with the expectation of further impedance rise over time (Figure 1).

**Figure 2:**
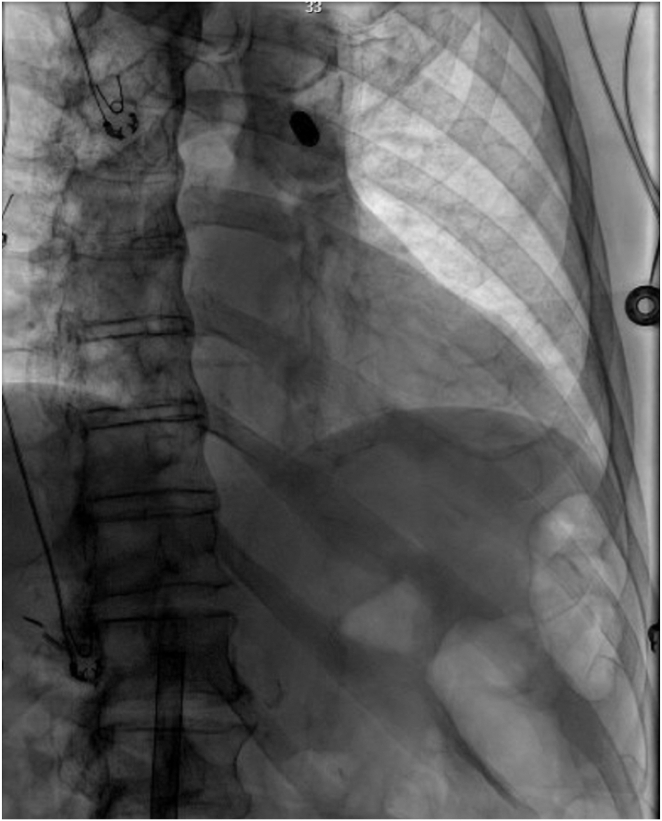
Leadless pacemaker device dislodgment into the left pulmonary artery.

**Figure 3:**
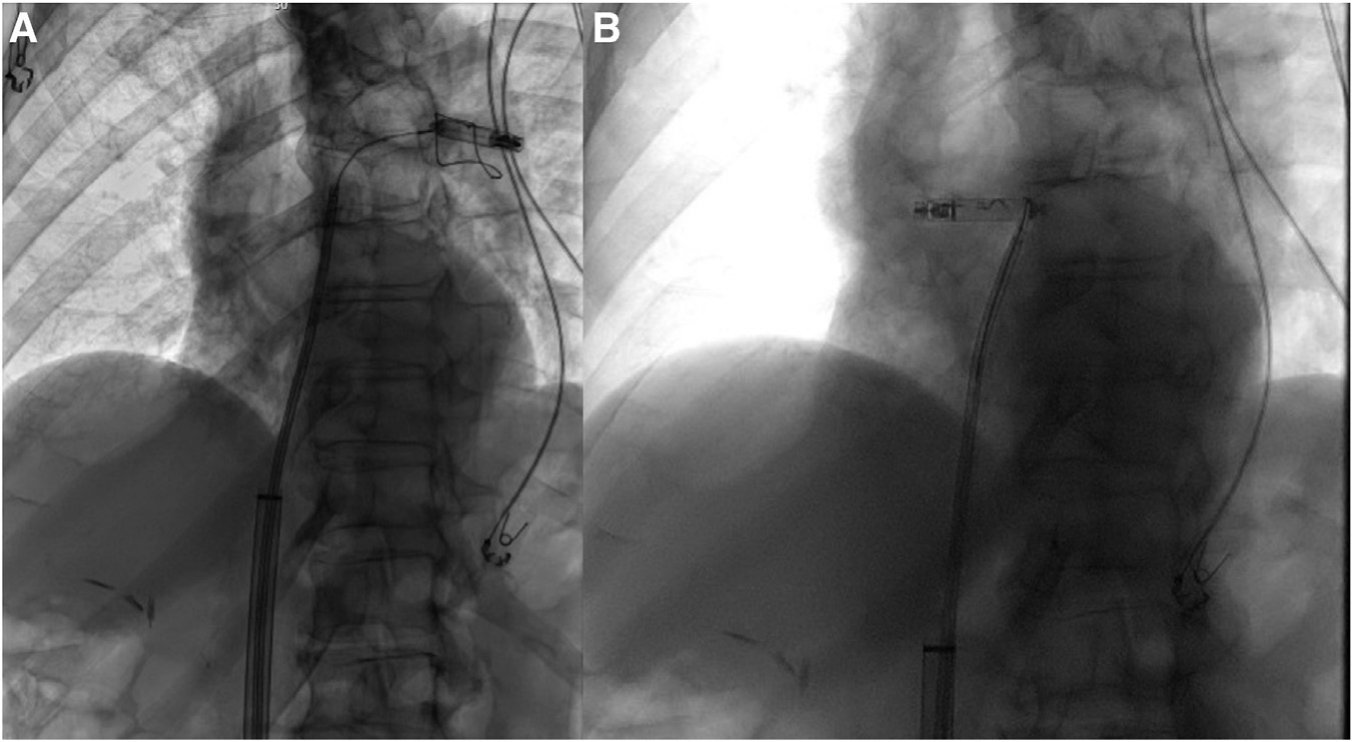
**A:** Leadless pacemaker device capture with a goose-neck snare. **B:** Leadless pacemaker device relocation into the right ventricular outflow tract.

After several minutes, loss of capture prompted fluoroscopy, revealing device dislodgment into the left pulmonary artery (Figure 2). Given our familiarity with the procedure, we opted to employ a sheath-and-snare technique for device extraction. This involved introducing a medium curve deflectable steerable sheath through the outer delivery sheath and advancing it into the right atrium using a 0.035 mm stiff wire. Subsequently, the stiff wire was exchanged with a deflectable mapping catheter to engage into the right ventricular outflow tract (RVOT) and the left pulmonary artery. After careful advancement of the medium curve deflectable sheath over the deflectable mapping catheter into the RVOT, a goose-neck snare was then substituted for the catheter (Figure 3A). The LP device was captured mid-body, pulled into the RVOT, but assumed a sideways position, lodging in the RVOT (Figure 3B). Retrieval was temporarily halted to prevent RVOT injury. The goose-neck snare was meticulously adjusted over the LP device with each heartbeat, sliding into the docking button, was shifted into a vertical position, and was pulled down into the right ventricular cavity (Supplemental Video 1).

Subsequently, the device was safely retrieved into the outer sheath and exteriorized from the femoral vein, and hemostasis was maintained by a figure-of-8 suture. A new device, implanted in the mid-interventricular septum using the standard technique, met appropriate deployment parameters: passing the device fixation test, R-wave sensing at 6.0 mV, right ventricular capture at 1.75 V at 0.5 ms, and an impedance of 1000 Ω. A transthoracic echocardiogram on the next day ruled out pericardial effusion, and device interrogation yielded normal results. In outpatient follow-up, the patient reported feeling well, with improvement in lightheadedness and no syncope. Device interrogation revealed normal parameters, and the patient expressed appreciation for the care received.

## Discussion

The AVEIR LP system first gained Food and Drug Administration approval in April 2022 to become the second actively implanted LP system. This system has proven implant success in 98%, safety in 96%, and effectiveness in 95.9% of 200 patients with attempted implantation in the LEADLESS II phase 2 trial.^1^ This helix-fixation LP system offers the ability to modify the delivery catheter and contact mapping before fixation, which can potentially improve the implantation success rate. There were 2 events (1%) of premature implantation and device migration in the LEADLESS II phase 2 trial.^1^ The LEADLESS II trial also reports 8 participants (4.1%) who did not meet the effectiveness criteria; 4 failed the capture threshold criteria and 4 failed the R-wave amplitude criteria, without reporting data on impedance change.^1^

Device impedance plays an important role in predicting device stability and pacing threshold on follow-up, especially in the setting of slightly elevated pacing threshold.^2^ A Spanish study has shown low pacing threshold on follow-up when initial threshold is <0.5 V at 0.24 ms or when initial threshold is >0.5 V at 0.24 ms in the setting of impedance >600 Ω among 110 passive-fixation LP implants (ie, MICRA^TM^).^3^ While much of the previous reported data on the relationship between acceptable impedance, device stability, and low pacing threshold has been published in patients with passive-fixation LP implants, the growing experience with helix-fixation LP implants is likely to follow the same rule.^2^^,^^3^ In a multicenter experience of 67 patients who underwent MICRA VR (41 patients) and AVEIR VR (26 patients) LP implant, immediate implant impedance was relatively similar between both devices and had averaged in the 768 ± 162 Ω in the AVEIR VR group.^4^ The goose-neck snare has repeatedly proven efficacy, safety, and cost-effectiveness in retrieval of intravascular foreign bodies.^5^ If specialized retrieval devices for dislodged implants fail, or if the provider prefers commonly used basic tools, our alternative method using sheath and snaring techniques offers a reliable solution.

## Conclusion

This case underscores the significance of verifying an acceptable impedance, suggestively close to 500 Ω, and monitoring impedance elevation along with establishing acceptable pacing threshold and R-wave sensing before safely releasing the AVEIR LP system during implantation. Additional future studies and case-based experiences are needed to further discover the appropriate safe implant impedance. We have also learned from this case that a simple goose-neck snare can be used to safely capture and retrieve the AVEIR LP over its docking button.

## Disclosures

All authors have no conflicts of interest to disclose.

## Appendix Supplementary Data

Supplemental Video legend Leadless pacemaker snaring and retrieval into the right ventricle after assuming a horizontal position in the right ventricular outflow tract.1
